# Biphasic Synaptic Ca Influx Arising from Compartmentalized Electrical Signals in Dendritic Spines

**DOI:** 10.1371/journal.pbio.1000190

**Published:** 2009-09-15

**Authors:** Brenda L. Bloodgood, Andrew J. Giessel, Bernardo L. Sabatini

**Affiliations:** 1 Department of Neurobiology, Harvard Medical School, Boston, Massachusetts, United States of America, 2 Howard Hughes Medical Institute, Harvard Medical School, Boston, Massachusetts, United States of America; Salk Institute for Biological Studies, United States of America

## Abstract

Excitatory synapses on mammalian principal neurons are typically formed onto dendritic spines, which consist of a bulbous head separated from the parent dendrite by a thin neck. Although activation of voltage-gated channels in the spine and stimulus-evoked constriction of the spine neck can influence synaptic signals, the contribution of electrical filtering by the spine neck to basal synaptic transmission is largely unknown. Here we use spine and dendrite calcium (Ca) imaging combined with 2-photon laser photolysis of caged glutamate to assess the impact of electrical filtering imposed by the spine morphology on synaptic Ca transients. We find that in apical spines of CA1 hippocampal neurons, the spine neck creates a barrier to the propagation of current, which causes a voltage drop and results in spatially inhomogeneous activation of voltage-gated Ca channels (VGCCs) on a micron length scale. Furthermore, AMPA and NMDA-type glutamate receptors (AMPARs and NMDARs, respectively) that are colocalized on individual spine heads interact to produce two kinetically and mechanistically distinct phases of synaptically evoked Ca influx. Rapid depolarization of the spine triggers a brief and large Ca current whose amplitude is regulated in a graded manner by the number of open AMPARs and whose duration is terminated by the opening of small conductance Ca-activated potassium (SK) channels. A slower phase of Ca influx is independent of AMPAR opening and is determined by the number of open NMDARs and the post-stimulus potential in the spine. Biphasic synaptic Ca influx only occurs when AMPARs and NMDARs are coactive within an individual spine. These results demonstrate that the morphology of dendritic spines endows associated synapses with specialized modes of signaling and permits the graded and independent control of multiple phases of synaptic Ca influx.

## Abstract 2

Dendritic spines compartmentalize synaptically-evoked biochemical signals. The authors show that electrical compartmentalization provided by a spine endows the associated synapse with additional modes of calcium signaling by shaping the kinetics of synaptic calcium currents.

## Author Summary

The vast majority of excitatory synapses in the mammalian central nervous system are made onto dendritic spines, small (< 1 fL) membranous structures stippled along the dendrite. The head of each spine is separated from its parent dendrite by a thin neck – a morphological feature that intuitively suggests it might function to limit the transmission of electrical and biochemical signals. Unfortunately, the extremely small size of spines has made direct measurements of their electrical properties difficult and, therefore, the functional implications of electrical compartmentalization have remained elusive. In this study, we use spatiotemporally controlled stimulation to generate calcium signals within the spine head and/or neighboring dendrite. By comparing these measurements we demonstrate that spines create specialized electrical signaling compartments, which has at least two functional consequences. First, synaptic stimulation, but not similar dendritic depolarization, can trigger the activation of voltage-gated calcium channels within the spine. Second, voltage changes in the spine head arising from compartmentalization shape the time course of synaptically evoked calcium influx such that it is biphasic. Thus, the electrical compartmentalization provided by spines allows for multiple modes of calcium signaling at excitatory synapses.

## Introduction

Various theories have been proposed to explain why excitatory synapses of principal neurons are formed onto the heads of dendritic spines. These include proposals that spines increase the maximal density of contacts onto a linear dendrite [1],[2], that the long spine increases the repertoire of possible presynaptic partners for each dendrite [3], and that the thin spine neck diffusionally isolates the associated postsynaptic terminal in order to allow the synapse-specific induction and expression of plasticity [4]. A further proposal that has been controversial is that the thin neck creates an electrical resistance that, in conjunction with the membrane capacitance, significantly filters synaptic potentials [4]–[7]. For example, a high spine neck resistance would allow for the generation of large synaptic potentials that are limited to the active spine head and result in differential activation of voltage-sensitive ion channels on either side of the neck [8]–[10].

Based on its morphology and diffusional properties, the electrical resistance of the neck of most dendritic spines in their basal state has been estimated to be <100 MΩ, too low to create a large voltage drop across the neck in response to typical synaptic currents as measured at the soma [1],[2],[11]. These estimates depend on an approximation of the resistivity of the intracellular space within the neck, which given the thinness of the structure, its high protein content, and our poor understanding of its biochemistry, may differ substantially from that of other portions of the cell. Furthermore, biophysical constraints of charge flow through a narrow juxtamembrane space may not be revealed by studies of diffusion of uncharged molecules. Lastly, current flow out of the neck into the dendrite may be attenuated even if the neck resistance is low due to opening of ion channels or transporters located selectively in the spine neck.

Several recent studies have suggested that electrical filtering by the spine neck may be substantial and may be regulated by activity. First, voltage-sensitive dye and second harmonic generation imaging have suggested shunting of synaptic currents at long-neck spines [5]. Similarly, glutamate uncaging at individual spines revealed that stimulation of spines with longer necks produces smaller potentials measured at the soma [5],[12],[13]. However, the lack of independent quantification of the number of AMPARs on these spines renders the interpretation of the data in these studies difficult. Second, diffusional equilibration across the spine neck is retarded following paired pre- and postsynaptic stimulation [14] or prolonged postsynaptic depolarization [9]. In such post-constriction spines the spine neck resistance may approach 1 GΩ, opening the possibility that activity-dependent regulation of spine/dendrite coupling is a mechanism for dynamic control of synaptic signals. Indeed, after apparent constriction of the spine neck by depolarization to 0 mV, the contribution of voltage-gated ion channels to synaptic Ca signaling is large; however, no analysis of synapses in their basal state was performed [9]. Lastly, a separate study of spines in their basal state found that VGCCs in the spine head are activated by unitary synaptic stimuli, suggesting the existence of a large synaptic depolarization within most spines. In particular, VGCCs blocked by SNX-482 are located in the spine head and open during synaptic stimuli, both contributing to synaptic Ca influx and leading to the activation of SK channels [14]. However, SNX-482-sensitive VGCCs are not found in the dendritic shaft, and therefore their selective activation in the spine head may reflect their inhomogeneous distribution and not compartmentalization of synaptic potentials.

Here we examine if the segregation of excitatory synapses onto the heads of dendritic spines endows the synapse with specialized modes of regulation and signaling that would not be available to synapses formed directly onto the dendritic shaft. Since quantifying the spine neck resistance and the voltage drop across the neck depends on difficult-to-measure cellular parameters, we avoid performing these estimates and instead focus on uncovering functional evidence that synaptic signals are influenced by the electrical and morphological properties of the spine. We find that, for spines in their basal state, the voltage drop across the spine neck during synaptic stimulation is sufficient to allow for differential activation of the voltage-sensitive ion channels in the spine and dendrite. Furthermore, depolarization reached in the spine during synaptic activity creates a large and brief component of Ca influx that is substantially accelerated compared to the typical slow kinetics of NMDAR signaling. The rapid and slow components of synaptic Ca influx arise through distinct mechanisms and are differentially affected by modulation of AMPARs and SK channels. Lastly, the biphasic nature of Ca influx arises directly from the colocalization of AMPARs and NMDARs on the spine head and cannot be recapitulated by stimulation in the adjacent dendrite.

## Results

**Figure 1:**
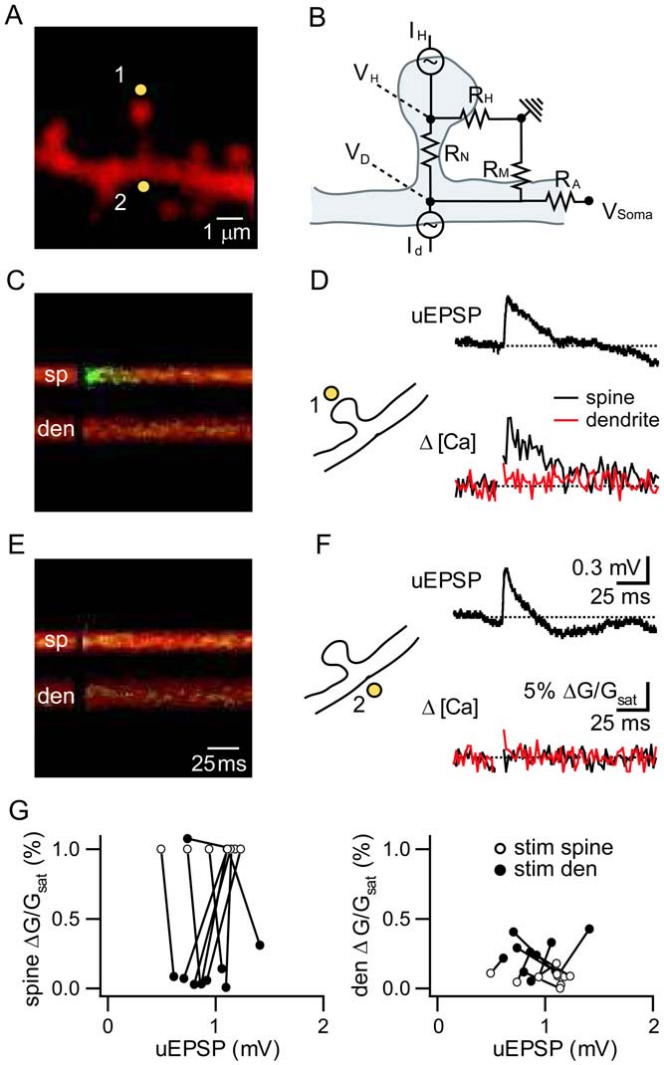
Differential activation of VGCCs over micron length scales. (A) Image of a spiny region of an apical dendrite of a CA1 hippocampal pyramidal neuron filled with the red, Ca-insensitive fluorophore Alexa Fluor-594 (5 µM) and the green, Ca-sensitive fluorophore Fluo-5F (150 µM). Yellow circles indicate uncaging locations at the spine (1) and dendrite (2). (B) Resistor network model of current flow through the spine neck. Current through open AMPARs enters the spine head (I_H_) and flows through both the spine head membrane (R_H_) and the spine neck (R_N_) resistors. The resistance of the neck creates a drop in voltage such that the voltage in the spine (V_H_) is greater than that in the dendrite (V_D_). Further filtering in the dendrite via membrane and axial resistances (R_M_ and R_A_, respectively) reduces the voltage measured at the soma (V_Soma_) such that it is less than V_D_. Alternatively, activating AMPARs in the dendrite (I_D_) generates a voltage difference such that V_D_ is greater than V_H_ while retaining the same relationship with V_Soma_. (C) Example of fluorescence collected in a line scan intersecting the spine (sp) and dendrite (den) shown in (A) during uncaging of MNI-glutamate near the spine head. Blocking glutamate-activated Ca sources reveals a small Ca signal mediated by VGCCs located on the spine head. (D) Corresponding uEPSP (*top*) measured at the soma and fluorescence transients (*bottom*) measured in the spine (black) and dendrite (red) in response to uncaging at the spine as shown in (C). (E) Example of fluorescence collected during line scans of the same spine and dendrite as in (C) during uncaging of MNI-glutamate near the dendrite at the base of the spine. (F) Corresponding uEPSP (*top*) measured at the soma and fluorescence transients (*bottom*) measured in the spine (black) and dendrite (red) in response to uncaging at the dendrite as shown in (E). (G) Δ*[Ca]_spine_* (*left*) and Δ*[Ca]_den_* (*right*) plotted as a function of uEPSP amplitude for each spine/dendrite pair analyzed. The amplitudes of Δ*[Ca]* are shown relative to Δ*[Ca]_spine_* measured when the spine was stimulated directly. Connected pairs show the data when stimulating the spine (white circles) and the dendrite (black circles).

We used 2-photon laser scanning microscopy (2PLSM) to monitor intracellular Ca transients and somatic whole-cell recordings to record potentials in mouse hippocampal CA1 pyramidal neurons in acute brain slices at near physiological temperatures (Figure 1). The red-fluorescing, space-filling fluorophore Alexa Fluor-594 and the green-fluorescing, Ca-sensitive fluorophore Fluo-5F were introduced into neurons through the somatic recording electrode. Controlled stimuli were delivered to spines and the neighboring portion of the dendrites with 2-photon laser photoactivation (2PLP) of caged glutamate using 500 µs pulses of 725 nm laser light (Figure 1A). Dendritic filtering was minimized by restricting analysis to spines located on proximal portions (<150 µm from the soma) of secondary apical dendrites.

### Voltage-Compartmentalization in Active Spines

A resistor network model of current flow in the spine and along the dendrite to the soma was used to predict the consequences of spine versus dendrite stimulation (Figure 1B). In this simple model, active AMPARs are represented as current sources and capacitance has been ignored. Current through open AMPARs enters the spine head (I_H_) and flows through both the spine head membrane (R_H_) and the spine neck (R_N_) resistors. The resistance of the neck creates a drop in voltage such that the voltage in the spine (V_H_) is greater than that in the dendrite (V_D_). Membrane and axial resistances of the dendrite (R_M_ and R_A_, respectively) further reduce the voltage at the soma (V_Soma_) such that it is less than V_D_. With current injection into the dendrite (I_D_), current will flow along the same path to the soma but in the opposite direction across the neck, thus maintaining the same relationship between V_D_ and V_Soma_ but resulting in V_H_<V_D_. If current is injected into the dendrite such that V_Soma_ is equal to that evoked by current injection into the spine, then the voltage profile from the base of the spine to the soma is the same in the two conditions. Therefore, differences observed in spine head VGCC activation following these two stimuli can be attributed to differences in the voltage between the base of the spine and the spine head and would indicate that the spine neck resistance R_N_ is sufficient to support a significant voltage drop.

In order to detect if synaptic stimuli lead to differential activation of VGCCs on either side of the spine neck, we measured glutamate uncaging-evoked Ca transients in the spine head (Δ*[Ca]_spine_*) and neighboring dendrite (Δ*[Ca]_den_*) while stimulating either the spine (Figure 1C and 1D) or the dendrite (Figure 1E and 1F). In this and all portions of the study, the uncaging laser power delivered to each spine was set in order to mimic the kinetics and amplitude of glutamate receptor activation following vesicular release using a photobleaching calibration strategy [15]. This stimulus results in ∼10–13 pA excitatory post-synaptic currents (EPSCs) and ∼0.8–1 mV potentials (EPSPs), consistent with the size of potentials generated by single active synapses associated with large-head spines studied here [16] and at the large end of the distribution of responses from all synapses [17],[18] formed onto proximal portions of apical dendrites of CA1 pyramidal neurons. When stimulating the dendrite, laser power was adjusted to evoke a similarly sized uncaging-evoked EPSP (uEPSP) as seen when the spine was stimulated directly. To isolate Ca influx through VGCCs, all glutamate-activated Ca sources were blocked using a combination of NMDAR, metabotropic glutamate receptor (mGluR), Ca-permeable AMPAR, and kainate receptor antagonists (CPP, MK801, MPEP, CPCCOEt, Joro spider toxin, NASPM, UBP302, respectively). SK and voltage-gated sodium channels were also blocked (with apamin and tetrodotoxin, respectively) to prevent nonlinear effects of these channels on synaptic signaling [15],[19],[20]. In these conditions, direct activation of the spine triggers a uEPSP as well as a rapidly rising and short-lived Δ*[Ca]_spine_* (Figure 1C and 1D). In contrast, stimulation of the dendrite near the base of the spine elicits a similarly sized uEPSP via activation of extrasynaptic AMPARs yet it produces negligible Δ*[Ca]_spine_* (Figure 1E and 1F). In this example neither stimulus produces significant Δ*[Ca]_den_*, although the magnitude of Δ*[Ca]_den_* was variable across experiments. On a spine-by-spine basis, stimulation of the dendrite reliably evoked Δ*[Ca]_den_*, but only in one case was able to evoke substantial Δ*[Ca]_spine_* compared to stimulating the spine head directly (Figure 1G). In two cases stimulation of the dendrite produced a sizable local Ca increase, possibly reflecting the presence of a small spine on the dendrite oriented along the optical axis of the microscope and thus beyond our ability to visually resolve.

**Figure 2:**
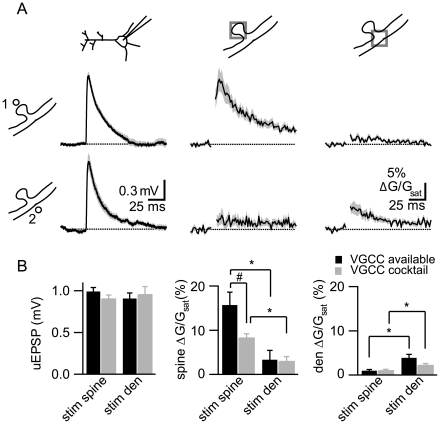
Synaptic, but not dendritic, depolarization activates VGCCs in the spine head. (A) uEPSPs recorded at the soma (*left*), Ca-dependent changes in fluorescence measured in the spine head (*middle*), and Ca-dependent changes in fluorescence measured in the dendrite (*right*) generated in response to uncaging at the spine (*top*) or dendrite (*bottom*). Data are shown as the mean (line)±SEM (shaded region). (B) Summary of amplitudes of uEPSPs (*left*), Δ*[Ca]_spine_* (*middle*), and Δ*[Ca]_den_* (*right*) evoked by spine or dendrite stimulation measured in control conditions (black) and in the presence of a cocktail of VGCC antagonists (grey). * indicates that the difference seen comparing spine stimulation versus dendrite stimulation is significant, whereas # indicates a significant difference comparing across control and VGCC cocktail conditions.

On average, dendrite and spine stimulation evoked equal amplitude uEPSPs as measured at the soma (0.99±0.05 mV, *n* = 15, and 0.91±0.07 mV, *n* = 10, respectively), whereas spine stimulation evoked ∼5-fold larger Δ*[Ca]_spine_* than dendritic stimulation (Δ**G/G_sat_: 15.7%±2.9% and 3.3%±2.1%, respectively) (Figure 2B). Both stimuli produced only small Δ*[Ca]_den_* (Δ**G/G_sat_: 0.9%±0.3% and 3.9%±0.8% for stimulation of the spine and dendrite, respectively) (Figure 2B). Thus, dendritic stimulation is unable to activate voltage-gated Ca sources located in the spine head.

To confirm that Δ*[Ca]_spine_* was mediated by VGCC activation, experiments were repeated in the additional presence of a cocktail of VGCC antagonists (nimodipine, ω-conotoxin-MVIIC, SNX-482, mibefradil, and nickel). Addition of these antagonists reduced Δ*[Ca]_spine_* (Δ**G/G_sat_: 8.2%±0.9% and 2.9%±1.1%, *n* = 7 for spine and dendrite stimulation, respectively) and Δ*[Ca]_den_* (Δ**G/G_sat_: 1.0%±0.3% and 2.2%±0.5%) without altering the uEPSP (0.90±0.05 mV and 0.95±0.10 mV) (Figure 2B). VGCCs resistant to this combination of antagonists have been reported and likely contribute the remainder of Δ*[Ca]_spine_* and Δ*[Ca]_den_* [21]–[23]. These results indicate the existence of a voltage drop across the spine neck that is sufficient to spatially compartmentalize activation of voltage-gated ion channels over a micron length scale.

### Biphasic Synaptic Ca Influx and Graded Control by AMPARs

**Figure 3:**
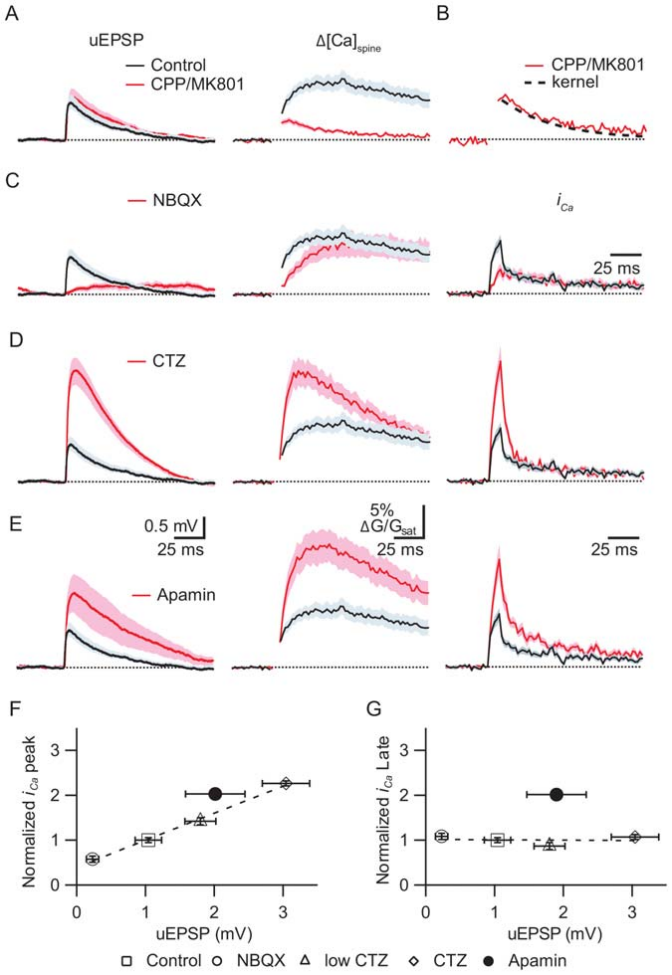
Synaptic AMPAR activation determines the early but not late phase of Ca entry into the spine. (A) uEPSPs (*left*) and Δ*[Ca]_spine_* (*right*) evoked in control conditions (black) or in the presence of the NMDAR antagonists CPP and MK801 (red). (B) uEPSP evoked Δ*[Ca]_spine_* measured in the presence of NMDAR antagonists (as in Panel A). Δ*[Ca]_spine_* is fit by a single exponential with *τ* = 42 ms (black dashed line), which is used as the deconvolution kernel throughout. (C) uEPSPs (*left*), Δ*[Ca]_spine_* (*middle*), and *i_Ca_* (*right*) in control conditions (black) and in the presence of the AMPAR antagonist NBQX (red). (D) uEPSPs (*left*), Δ*[Ca]_spine_* (*middle*), and *i_Ca_* (*right*) in control conditions (black) and in the presence of 5.0 µM CTZ to accentuate AMPAR opening (red). (E) uEPSPs (*left*), Δ*[Ca]_spine_* (*middle*), and *i_Ca_* (*right*) in control conditions (black) and in the presence of the SK channel antagonist apamin (red). (F and G) The amplitude of the peak (F) and prolonged phase of *i_Ca_* (G) plotted as a function of uEPSP amplitude for control conditions, AMPAR blockade (NBQX), AMPAR enhancement (low CTZ and CTZ), and SK channel blockade (apamin).

In order to determine if the depolarization reached in the spine during synaptic activity shapes synaptic signals, we examined if the opening of AMPARs, which provide the bulk of current influx that produces the EPSP, secondarily alters the magnitude or kinetics of Ca current into the spine (*i_Ca_*) (Figure 3). Previous studies have reported variable effects of blocking AMPARs on the peak of synaptically evoked Ca transients [9],[24],[25]. However, in fluorescence imaging of Ca transients, the time course of *i_Ca_* is obscured by the presence of the exogenous Ca indicator, which slows the clearance of Ca from the spine. In the absence of exogenous buffer, the low (∼25) endogenous Ca buffer capacity of the apical spines of CA1 pyramidal neurons allows spine head Ca to closely follow the kinetics of opening of Ca sources [26]. To determine the time course of *i_Ca_*, we corrected for the kinetics of Ca handling by performing a deconvolution with the impulse response of Ca handling of the spine (see methods) [26],[27]. The impulse response was estimated from the decay of the synaptically evoked fluorescence transient measured in the presence of NMDAR antagonists, which is generated by Ca influx that is impulse-like relative to the decay kinetics of Δ*[Ca]_spine_*. Blockade of NMDARs with CPP/MK801 had no significant effect on uEPSP amplitudes (1.04±0.19 mV, *n* = 18, and 1.23±0.23 mV, *n* = 17, control and CPP/MK801, respectively) but reduced the early phase (20–50 ms post-uncaging) of Δ*[Ca]_spine_* ∼60% (early Δ**G/G_sat_: 9.48%±1.12% and 4.09%±0.62% in control and CPP/MK801; *p*<0.05) while nearly eliminating the later portion (50–120 ms post-uncaging) (late Δ**G/G_sat_: 8.48%±1.3% and 0.90%±0.16%; *p*<0.05) (Figure 3A). These results confirm the dominant role of Ca influx through NMDARs in generating synaptically evoked Ca transients and, in particular, in mediating the prolonged phases of synaptic Ca influx. The remaining spine Ca transient was well described by a single exponential decay with a time constant of ∼42 ms, which was used as the Ca impulse response in deconvolution analysis below.

Blockade of AMPARs with NBQX largely eliminated the uEPSP (0.23±0.09 mV, *n* = 11, *p*<0.05 versus control) (Figure 3C) and decreased the initial portion of Δ*[Ca]_spine_* (early Δ**G/G_sat_: 7.24%±1.5%, *p*<0.05 versus control) without significant effect on the prolonged phase (late Δ**G/G_sat_: 7.99%±1.70%). Deconvolution of the spine head fluorescence transients in control conditions and in the presence of NBQX reveals a large rapid phase of Ca influx that lasts ∼10 ms and that is eliminated by AMPAR blockade (Figure 3C). A similar rapid and AMPAR-dependent phase of Ca influx is also evoked by briefer glutamate uncaging pulses (300 µs) that elicit smaller uEPSPs (0.42±0.06 mV; *n* = 15) (Figure S1). These results indicate that, in addition to providing the depolarization that underlies the EPSP, AMPAR activation transiently boosts a rapidly activating Ca source in the spine that dominates the early phase of synaptic Ca transients.

In a converse set of experiments, AMPAR opening was enhanced using cyclothiazide (CTZ) (Figure 3D), which prevents AMPAR desensitization and increases the affinity of the receptor for glutamate [28]. We found that low levels of CTZ (2.5 and 5 µM) increased the amplitude of the uEPSP in a graded manner (Figure 3D, 3F, and 3G) (uEPSP: 1.80±0.23 mV, *n* = 17, and 3.04±0.34 mV, *n* = 7, for 2.5 and 5 µM, respectively). In addition, CTZ increased the amplitude of the early phase of Δ*[Ca]_spine_* (early ΔG/G_sat_: 11.2%±3.0% and 16.91%±1.89%, respectively) with no significant effect on the prolonged phases (late ΔG/G_sat_: 8.2%±2.3% and 11.27%±1.33%). Deconvolution analysis revealed that CTZ selectively boosted the amplitude of the rapid phase of *i_Ca_*, consistent with a modulation of spine potential and Ca influx during this period of depolarization. The prolonged phase of Ca influx is independent of AMPAR opening and is consistent with Ca influx through NMDARs in a spine that has returned to resting potentials. The lack of modulation of this phase is an independent confirmation that the degree of glutamate uncaging did not vary across conditions.

If the rapid phase of *i_Ca_* reflects Ca influx during a short-lived depolarization in the spine, its magnitude or duration should be increased by manipulations that slow the repolarization of the spine following synaptic stimuli. We have previously described that SK channels present in the spine are activated by Ca_V_2.3 type VGCCs and act to negatively regulate synaptic Ca influx [15]. Here we find that application of the SK antagonist apamin boosted the amplitude of the uEPSP (2.01±0.43 mV, *p*<0.05 versus control) in a manner similar to CTZ but, in contrast to CTZ, increased both the early and sustained phases of Δ*[Ca]_spine_* (early and late ΔG/G_sat_: 20.18%±2.53% and 16.59%±2.23%, respectively; *p*<0.05 for each versus control) (Figure 3E). Deconvolution revealed a larger and more prolonged rapid *i_Ca_* as well as an increase in its late phase. Although SK and AMPAR modulation both proportionally regulate the peak amplitudes of *i_Ca_* and the uEPSP, only apamin increases the amplitude of the prolonged phase of *i_Ca_* (Figure 3F). These data confirm that the rapid phase of *i_Ca_* is not due to direct Ca influx through AMPARs since increasing spine depolarization without altering the number of open AMPARs reduces the driving force for Ca influx and would be predicted to decrease Ca influx through these receptors. These results are consistent with SK channels opening rapidly following synaptic stimulation and repolarizing the spine, thereby directly truncating the synaptic potential in the spine, indirectly terminating the rapid phase of synaptic Ca influx, and reducing the prolonged phase of Ca entry through NMDARs [15],[20]. Thus, AMPARs and SK channels, which modulate the amplitude and kinetics of the synaptic potential in the spine, also regulate the kinetics of synaptically evoked Ca currents and spine Ca transients, indicating a functional role of spine depolarization in shaping synaptic signals.

### Requirement for Colocalization of AMPARs and NMDARs

**Figure 4:**
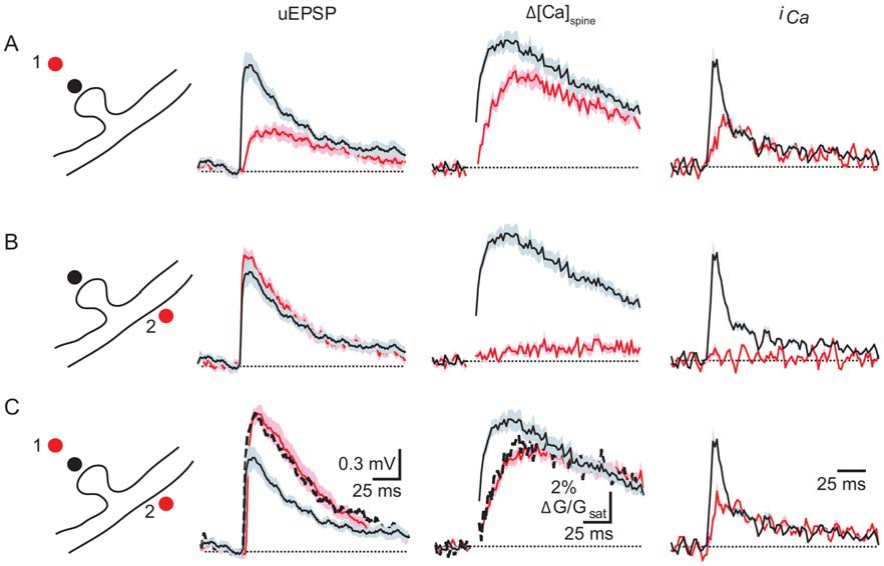
AMPARs and NMDARs must be colocalized to enhance the early phase of synaptic Ca influx. (A) Schematic of uncaging locations (*far left*): control is in black and “NMDAR-only” distant uncaging is illustrated in red. uEPSPs (*left*), Δ*[Ca]_spine_* (*middle*), and *i_Ca_* (*right*) are shown for control (black) and “NMDAR-only” (red) uncaging locations. (B) Schematic of uncaging locations (*far left*): control is in black and “AMPAR-only” dendritic uncaging is illustrated in red. uEPSPs (*left*), Δ*[Ca]_spine_* (*middle*), and *i_Ca_* (*right*) are shown for control (black) and “AMPAR-only” (red) uncaging locations. (C) Schematic of uncaging locations (*far left*): control is in black and the paired NMDAR-only and AMPAR-only uncaging locations are illustrated in red. uEPSPs (*left*), Δ*[Ca]_spine_* (*middle*), and *i_Ca_* (*right*) are shown for control (black) and paired NMDAR-only and AMPAR-only (red) uncaging locations. The linear sums of the responses obtained with NMDAR-only and AMPAR-only uncaging protocols shown in Panels (A) and (B) are indicated by the dashed lines.

In order to understand if the graded modulation of synaptic Ca influx by AMPAR opening is made possible by the electrical properties of the spine neck, we examined if the opening of dendritic AMPARs located at the base of the spine is also able to enhance the rapid phase of *i_Ca_* (Figure 4). For this analysis, each spine was stimulated with four different spatiotemporal patterns of glutamate uncaging designed to mimic (1) normal synaptic activation of AMPARs and NMDARs on the spine head, (2) activation of only NMDARs on the spine head, (3) activation of only AMPARs on the neighboring dendrite, and (4) near simultaneous activation of spine head NMDARs and dendritic AMPARs.

As expected, stimulation of the spine in order to locally open both AMPARs and NMDARs resulted in a robust uEPSP (1.1±0.13 mV, *n* = 11) and Δ*[Ca]_spine_* (early and late ΔG/G_sat_: 8.28%±0.64% and 6.31%±0.58%) (Figure 4A). In order to separate AMPAR and NMDAR activation, we took advantage of the high affinity of NMDARs for glutamate [29] and the existence of extrasynaptic dendritic AMPARs [30]–[32]. Glutamate uncaging at a spot located ∼1.5–3 microns from the spine head for 2 ms releases glutamate that activates spine NMDARs but, because of the rapid fall of glutamate concentration from the uncaging location, is insufficient to activate AMPARs. Consistent with preferential activation of spine NMDARs, this stimulus results in a small uEPSP (0.40±0.05 mV, *n* = 11) and Δ*[Ca]_spine_* (early and late ΔG/G_sat_: 5.85%±0.40% and 5.07%±0.40%, respectively) (Figure 4A) that is similar to that measured in the presence of AMPAR antagonists. Furthermore, in a separate set of experiments, the glutamate receptor dependence of the spine head calcium transient generated by this stimulus (early and late ΔG/G_sat_: 5.05%±0.63% and 4.54%±0.55%, respectively; *n* = 8) was analyzed and found to be blocked by CPP (early and late ΔG/G_sat_: 0.35%±0.29% and 0.48%±0.23%, respectively; *n* = 6) and insensitive to NBQX (early and late Δ**G/G_sat_: 5.20%±0.90% and 4.17%±0.84%, respectively; *n* = 9), confirming that it is mediated by NMDARs with little influence from AMPARs. Although this stimulus also generates a small uEPSP, it is referred to here as the “NMDAR only” stimulus for convenience.

In order to supply AMPAR-dependent depolarization without direct stimulation of spine NMDARs, we applied a stimulus to the base of the spine as in Figure 1 that activates dendritic AMPARs and produces a uEPSP of similar amplitude (1.19±0.06 mV) to direct stimulation of the spine but only minimal Δ*[Ca]_spine_* (early and late Δ**G/G_sat_: 0.62%±0.13% and 0.87%±0.18%, respectively) (Figure 4B). Failure of this “AMPAR only” stimulus to induce Ca influx into the spine head is due to both a failure of the depolarization to activate spine head VGCCs and a failure of glutamate to diffuse to and significantly activate spine NMDARs (Figure 4B). Lastly, we paired the AMPAR-only and NMDAR-only stimuli and compared these to results obtained when both channel types are spatially co-activated by direct stimulation of the spine. We find that the temporally paired but spatially separated activation of AMPARs and NMDARs results in a uEPSP (1.57±0.15 mV) and Δ*[Ca]_spine_* (early and late Δ**G/G_sat_: 5.93%±0.38% and 5.80%±0.44%, respectively) that are predicted by the linear sums of the NMDAR-only and AMPAR-only uEPSP and Δ*[Ca]_spine_* (Figure 4C). However, despite the larger amplitude of the uEPSP generated by this paired stimulus, it failed to enhance the rapid phase of Ca influx beyond that seen with NMDAR-only stimulation. Deconvolution analysis reveals that the AMPAR-only, NMDAR-only, and paired stimulus all failed to generate the spike-like rapid phase of Ca influx seen with direct stimulation of the spine. Thus, open AMPARs must be colocalized with NMDARs on the spine in order to enhance and accelerate synaptic Ca influx.

## Discussion

We have demonstrated that synaptic depolarization is sufficient to activate voltage-sensitive Ca sources located on the spine but that an EPSP-like dendritic depolarization is not. Previous studies have shown that synaptically evoked Ca influx is limited to the active spine but were performed in conditions in which the contribution of VGCCs to Ca influx was obscured [33],[34]. These studies had been designed to measure NMDAR-mediated Ca influx and either held the neuron at 0 mV to intentionally prevent stimulus-evoked VGCC activation, or imaged using a high affinity Ca buffer, which enhances the contributions of long-lived Ca sources such as the NMDAR relative to those of short-lived Ca sources such as VGCCs. Our study demonstrates that under conditions in which Ca influx through spine VGCCs can be measured, their activation only occurs when they are located on the active spine. This indicates that VGCCs and other voltage-gated channels in one spine are unlikely to be opened by synaptic activity at neighboring spines or subthreshold depolarizations in the dendrite. Since specializations of Ca handling prevent diffusion of Ca from active to inactive spines [26] and electrical filtering prevents the spread of VGCC-activating potentials, signaling cascades triggered by synaptic activation of VGCCs are limited to the active spine. Thus, synaptic cross-talk among neighboring spines is likely to be predominately biochemical in nature and downstream of Ca [35],[36]. It is important to note that these conclusions do not apply to stimuli that trigger local or back-propagating dendritic action potentials, which reliably invade active and inactive spines [17],[21],[34],[37],[38].

In addition, we find that the existence of a large depolarization within the active spine transiently couples AMPAR activation and synaptic Ca sources, generating a fast, spike-like phase of Ca influx. This rapid phase is temporally and mechanistically distinct from the prolonged phase of NMDAR-dependent Ca influx. Since the activation of biochemical events downstream of Ca depend on both the kinetics and amplitude of the Ca transients, independent modulation of these parameters may fine-tune the activation of Ca-dependent signaling pathways. Furthermore, differential regulation of each phase may permit subtle and nuanced regulation of synaptic signaling cascades, such as those underlying spike-timing dependent potentiation and depression.

The current study was performed using combined 2PLSM and 2PLP, which allowed delivery of controlled stimuli to visually identified portions of the dendritic arbor [17],[35],[39],[40]. This approach also allows the examination of postsynaptic signaling in conditions, such as blockade of voltage-gated Na and Ca channels, which prevent action potential-evoked release of neurotransmitter from the presynaptic terminal. When using glutamate uncaging, an important parameter that must be carefully considered is the strength at which individual postsynaptic terminals are stimulated and how the amount of glutamate released compares to that contained in a single vesicle. Here we calibrate uncaging laser power delivered to each spine using a photo-bleaching strategy [15]. This approach delivers an amount of glutamate that, on average and under conditions of excellent space-clamp, produces a uncaged-evoked EPSCs of ∼10–13 pA in amplitude, similar to that previously reported for spontaneous miniature EPSCs in hippocampal pyramidal neurons [18],[41],[42]. In current clamped neurons, this same stimulus results in ∼0.8–1 mV uEPSPs (see Figure 2) [15]. This amplitude is similar to that of unitary EPSPs in 2- to 4-wk-old rat tissue evoked by stimulation of individual synapses formed onto large dendritic spines similar to those studied here [16]. In contrast, a study of adult (6–10 wk) rat hippocampal CA1 pyramidal neurons found that single vesicle EPSPs recorded from proximal dendrites and measured in Sr2+ or following hypertonic sucrose puffs averaged 0.2 mV in amplitude and ranged from 0.1 to 1.5 mV while EPSCs ranged from a few to tens of picoamps [18]. The large amplitude of uEPSPs in our hands despite the relatively small size of uncaged-evoked EPSCs may result from the smaller size and hence larger input resistance of juvenile mouse CA1 pyramidal neurons. In addition, in this study we selected large-head spines, which have higher AMPA receptor content compared to the average of all synapses found on the apical dendrite and are thus expected to generate larger potentials [43],[44].

In order to ensure that our results are not due to overly strong stimulation, we analyzed the kinetics and NBQX sensitivity of spine head Ca transients evoked by smaller depolarizations (∼0.4 mV) evoked by uncaging for 300 µs. This smaller stimulus also produced biphasic calcium influx with a rapid phase that was blocked by antagonists of AMPARs (Figure S1). Taken together, these points suggest that while the uncaging stimulus is at the upper end of the physiologically relevant range, biphasic Ca influx occurs over a spectrum of relevant stimulus strengths. However, one cannot fully discard the possibility that the spatiotemporal pattern of glutamate release following uncaging enhances effects that contribute less robustly during release of glutamate from the presynaptic terminal.

Our results predict that changes in the numbers of AMPARs at the synapse over the course of development or following the induction of many forms of plasticity will not only determine the amplitude of the synaptic potential but will also directly alter the synaptic Ca transient. Finally, the morphological features of the spine, specifically the dimensions of the spine neck, can restrict the movement of molecules [11],[14],[45] and attenuate electrical signals exchanged between the spine head and dendrite [5]. Thus, identification of molecules involved in regulating the length and, in particular, the diameter of the spine neck will be important future avenues of study.

## Materials and Methods

### Animal Handling and Slice Preparation

Animals were handled according to protocols that have been approved by the Harvard Standing Committee on Animal Care and are in accordance with National Institutes of Health guidelines. Transverse hippocampal slices were prepared from C57/Blk6 mice from P15–P18 as described previously [15]. Animals were anesthetized by inhalation of isoflurane. The cerebral hemispheres were quickly removed and placed into cold choline-based artificial cerebrospinal fluid (choline-ACSF) containing 25 mM NaHCO_3_, 1.25 mM NaH_2_PO_4_, 2.5 mM KCl, 7 mM MgCl_2_, 25 mM glucose, 1 mM CaCl_2_, 110 mM choline chloride, 11.60 mM ascorbic acid, and 3.10 mM pyruvic acid, and equilibrated with 95%O_2_/5%CO_2_. Tissue was blocked and transferred into a slicing chamber containing choline-ACSF. Transverse hippocampal slices (300 µm) were then cut with a Leica VT1000S (Leica Instruments, Nussloch, Germany) and transferred into a holding chamber containing ACSF consisting of (in mM) 127 NaCl, 2.5 KCl, 25 NaHCO_3_, 1.25 NaH_2_PO_4_, 2.0 CaCl_2_, 1.0 MgCl_2_, and 25 glucose, equilibrated with 95%O_2_/5% CO_2_. Slices were incubated at 32°C for 30–45 min and then left at room temperature until recordings were performed.

### Electrophysiology

Whole-cell recordings were made from CA1 pyramidal neurons visualized under infrared differential interference contrast microscopy (IR-DIC). Patch pipettes (open pipette resistance 2.5–4.5 MΩ) were filled with an internal solution containing (in mM) 140 KMeSO_4_, 8 NaCl, 1 MgCl_2_, 10 HEPES, 5 MgATP, and 0.4 Na_2_GTP (pH 7.3). In the first set of experiments (Figures 1 and 2), 150 µM Fluo-5F (Molecular Probes, K_D_∼1.1 µM) and 5 µM Alexa Fluor-594 were included in the internal solution; in the remaining experiments (Figures 3 and 4), 300 µM Fluo-5F and 10 µM Alexa Fluor-594 were used. Recordings were made with an Axoclamp 200B or Multiclamp 700B amplifier (Axon Instruments, Union City, CA, USA). Data were filtered at 5 kHz and sampled at 10 kHZ. Cells were held at −65 mV in voltage-clamp mode, and DC current was injected to hold cells at approximately −65 mV in current-clamp mode. Cells were rejected if holding currents exceed −50 pA. Series and input resistance were measured throughout the experiment, and recordings were discarded if series resistance exceeded 20 MΩ. All recordings were done at 32°C and within 7 h of slice preparation. D-serine was included in the ACSF in all recordings to reduce NMDAR desensitization.

### Pharmacology

Pharmacological agents were used at the following final concentrations as indicated in the text (in µM): 10 D-serine (Sigma-Aldrich, St. Louis, MO, USA), 2.5 or 5 CTZ (Tocris Biosciences, Ellisville, MO, USA), 0.1 apamin (Calbiochem, La Jolla, CA, USA), 20 CPP (Tocris), 40 MK-801 (Tocris), 10 NBQX (Tocris), 25 UBP302 (Tocris), 10 NASPM (Tocris), 0.5 Joro spider toxin (Sigma-Aldrich), 1 MPEP (Tocris), 100 CPCCOEt (Tocris), 1 tetrodotoxin (Tocris), 20 nimodipine (Sigma-Aldrich), 1 ω-conotoxin-MVIIC (Peptides International, Louisville, KY, USA), 0.3 SNX-482 (Peptides International), 10 mibefradil (Sigma-Aldrich), and 50 nickel (Sigma-Aldrich). Ca-permeable AMPARs are expressed in synapses of CA1 pyramidal neurons only in a narrow time window following LTP induction [46] or homeostatic plasticity [47],[48]. Nevertheless, for the analysis in Figure 1 we included antagonists that we previously have shown block Ca influx through these channels in spines of other neuron classes [39],[49].

### Combined 2PLSM and 2PLP

Custom built 2-photon laser scanning microscopes based on BX51Wl microscopes (Olympus) were used as described previously [39]. Ti-sapphire lasers (Mira/Verdi, Coherent) tuned to 840 and 725 nm were used for imaging and glutamate uncaging, respectively. In all uncaging experiments, 3.75 mM MNI-glutamate (Tocris) was included in a small volume (∼9 ml) of re-circulating ASCF. Unless otherwise stated in the text, uncaging laser pulse duration was 0.5 ms and power delivered to each spine was standardized to bleach ∼50% of the red fluorescence in the spine head as described [15]. This corresponded to uncaging laser power as measured at the focal plane of the back aperture of the objective of ∼35 mW, similar to that used in other 2P-uncaging studies [36]. Image and electrophysiology acquisition was controlled by custom software written in MATLAB (Mathworks).

### [Ca] Measurements and Deconvolution Analysis

Cells were filled with two fluorescent dyes: a Ca-insensitive fluorophore (Alexa Fluor-594), which fluoresces in the red (collected via 600–660 interference filter), and a Ca-sensitive fluorophore (Fluo-5F), which fluoresces in the green (collected via 500–550 interference filter). Red fluorescence was used to identify spines and dendrites. For a given stimulus, changes in fluorescence were quantified as: ΔG/R(t) = (F_green_(t)−F_rest,green_)/(F_red_−I_dark, red_). F_green_(t) is the green fluorescence signal as a function of time, F_rest,green_ is the green fluorescence before stimulation. I_dark, red_ is the dark current in the red channel. ΔG/R was measured in saturating Ca (G_sat_/R) for each dye combination and batch of intracellular solution by imaging a sealed pipette with equal volumes intracellular solution and 1 M CaCl_2_. ΔG/R measurements from the spine were divided by G_sat_/R, yielding the reported measures of ΔG/G_sat_. This value is independent of the collection efficiencies of red and green photons and should be directly comparable across laboratories.

In order to estimate the time course of synaptic Ca influx, synaptic Ca transients were deconvolved using the impulse response kernel derived as follows. Spines were stimulated with 2-photon uncaging in the presence of CPP/MK801 to block NMDARs. The resulting spine ΔG/G_sat_ transients are well fit by a single exponential with a time constant of 42 ms (see Figure 3B). Since this represents the response to an impulse-like synaptic stimulus that is much briefer than the kinetics of Ca clearance, it is a good approximation of the impulse response and can be used as a deconvolution kernel. In order to perform the deconvolution, ΔG/G_sat_ transients were smoothed using a 3- or 5-point box smoothing algorithm. The Fourier transforms of both the smoothed transients and the kernel were computed, and the transform of the fluorescence transient was divided by that of the kernel. The inverse Fourier transform of the resulting trace was computed, yielding a trace that is proportional to Ca current and that reports the time course of Ca influx. This deconvolution approach assumes linearity of Ca handling once it has entered the spine and uses the time course of fluorescence transients to extract the time course and the relative amplitude of Ca current. This approach does not require linear activation of Ca channels and is robust in the face of nonlinear interactions between Ca sources. In the presence of Fluo-5F, Ca handling within the spine is linear and stimulus-evoked Ca transients are well described by the convolution of the time course of Ca influx and an exponential impulse response [26],[27].

### Data Analysis and Statistics

Off-line data analysis was performed using custom software written in Igor Pro (Wavemetrics) and MATLAB (Mathworks). The early and late phases of the Ca transient were calculated by averaging 20–50 ms and 50–120 ms post-uncaging, respectively. The early and late phases of *i_Ca_* were calculated by using the peak and the average from 10–110 ms post-uncaging, respectively. All data are expressed as the mean±SEM. In the figures, average traces are shown as the mean (line)±the SEM (shaded regions). A two-tailed *t*-test was used to determine significance of differences in uEPSP and ΔG/G_sat_ across conditions. *p*<0.05 was considered significant.

## Supporting Information

Figure S1 **Biphasic Ca influx is also evoked by small uEPSPs.** uEPSPs (*left*), Δ*[Ca]_spine_* (*middle*), and *i_Ca_* (*right*) evoked by uncaging with 300 µs laser pulses in control conditions (black, *n* = 18) and in the presence of NBQX (red, *n* = 15). Although the amplitudes of all three signals are decreased compared to those evoked by 500 µs laser pulses (Figure 3), the rapid and NBQX-sensitive phase of Ca influx is preserved.
(0.67 MB EPS)

## Abbreviations

2PLP: 2-photon laser photoactivation
2PLSM: 2-photon laser scanning microscopy
AMPAR: AMPA-type glutamate receptor
CTZ: cyclothiazide
mGluR: metabotropic glutamate receptor
NMDAR: NMDA-type glutamate receptor
uEPSP: uncaging-evoked EPSP
VGCC: voltage-gated Ca channel
